# Vitamin D as a follicular marker of human oocyte quality and a serum marker of in vitro fertilization outcome

**DOI:** 10.1007/s10815-018-1179-4

**Published:** 2018-05-17

**Authors:** Przemysław Ciepiela, Antoni J. Dulęba, Ewelina Kowaleczko, Kornel Chełstowski, Rafał Kurzawa

**Affiliations:** 1 Department of Gynecology and Reproductive Health, Pomeranian Medical University, 48 Żołnierska St., 71-210 Szczecin, Poland; 2 VitroLive Fertility Clinic, 2A Kasprzaka St., 71-074 Szczecin, Poland; 3 Division of Reproductive Endocrinology and Infertility, University of California, San Diego, 3350 La Jolla Village Dr, San Diego, CA 92161 USA; 4 Department of Laboratory Diagnostics and Molecular Medicine, Pomeranian Medical University, 72 Powstańców Wielkopolskich Ave., 70-111 Szczecin, Poland

**Keywords:** Oocyte quality, Vitamin D [25(OH)D], Follicular fluid, ICSI (intracytoplasmic sperm injection), Single embryo transfer (SET)

## Abstract

**Purpose:**

This study investigated the relationship between the vitamin D [25(OH)D] level in individual follicles and oocyte developmental competence.

**Methods:**

A prospective cohort study in a private infertility center. Infertile women (*N* = 198) scheduled for intracytoplasmic sperm injection (ICSI) and a single embryo transfer (SET) provided serum samples and 322 follicular fluid (FF) specimens, each from a single follicle on the day of oocyte retrieval.

**Results:**

FFs corresponding to successfully fertilized oocytes (following ICSI) contained significantly lower 25(OH)D level compared with those that were not fertilized (28.4 vs. 34.0 ng/ml, *P* = 0.001). Top quality embryos on the third day after fertilization, when compared to other available embryos, developed from oocytes collected from follicles containing significantly lower 25(OH)D levels (24.56 vs. 29.59 ng/ml, *P* = 0.007). Positive hCG, clinical pregnancy, and live birth rates were achieved from embryos derived from oocytes that grew in FF with significantly lower 25(OH)D levels than in follicles not associated with subsequent pregnancy. The concentration of 25(OH)D in FF in women with negative hCG was 32.23 ± 20.21 ng/ml, positive hCG 23.62 ± 6.09 ng/ml, clinical pregnancy 23.13 ± 6.09 ng/ml, and live birth 23.45 ± 6.11 ng/ml (*P* < 0.001). Women with serum 25(OH)D < 20 ng/ml had not only a higher fertilization rate (71 vs. 61.6%, *P* = 0.026) and a higher clinical pregnancy rate (48.2 vs. 25%, *P* = 0.001), but also higher miscarriage rate (14.5 vs. 3.8%, *P* = 0.013) compared with those with levels ≥ 20 ng/ml.

**Conclusion:**

This study reveals that the level of 25(OH)D in FF correlates negatively with the oocytes’ ability to undergo fertilization and subsequent preimplantation embryo development. Oocytes matured in FF with low 25(OH)D concentration are more likely to produce top quality embryos and are associated with higher pregnancy and delivery rates. On the other hand, low serum vitamin D concentration is associated with higher miscarriage rates.

## Introduction

Traditionally, recognized actions of vitamin D include calcium and phosphorus homeostasis, as well as the support of bone mineralization, while its deficiency leads to an increased risk of osteoporosis, bone fractures, and muscle weakness [1]. However, there is growing evidence that 25-hydroxyvitamin D [25(OH)D] exerts many other important actions, especially on human reproductive functions [2]. Vitamin D can contribute to the restoration of the menstrual cycle and endometrial proliferation [3, 4], promote the development of follicles [5], ameliorate primary dysmenorrhea [6], and reduce the occurrence of uterine fibroids [7]. Vitamin D receptors (VDR) have been identified in the female reproductive tissues including human ovarian, endometrial and fallopian tube epithelial cells, placenta, and decidual cells [8]. Vitamin D via its receptor can modulate ovarian steroidogenesis [9]. Expression of VDR is increased during pregnancy. Additionally, data from clinical reports suggest that lower vitamin D level is associated with gestational diabetes and preeclampsia [10, 11].

However, the relationship of vitamin D levels with IVF outcomes remains controversial. Although Polyzos et al. [12] reported that women with deficient serum vitamin D had a lower clinical pregnancy rate compared to women with normal levels of vitamin D, other studies led to opposite conclusions [13–18]. Because of these conflicting reports, and given that vitamin D deficiency is not correlated with ovarian stimulation characteristics or with markers of embryo quality [15, 19], it has been postulated that vitamin D deficiency may negatively affect pregnancy rates in IVF with an effect mediated through the endometrium [12, 15, 20]. In both the epithelial and stromal cells of the endometrium, vitamin D induces and upregulates the transcription of HOXA 10, a gene essential for implantation with increased expression during the window of implantation [21, 22]. Conversely, when a transfer of synchronous euploid blastocyst is performed, the vitamin D status does not impact the outcomes [14].

To date, published studies on vitamin D and assisted reproduction technologies (ART) have reported outcomes related to clinical pregnancy rates or live birth rates. However, these studies were not designed to evaluate the direct relationship of local-intrafollicular vitamin D levels with the ability of the individual oocyte to undergo fertilization and subsequent embryo development [13–15, 18, 23, 24]. The present prospective study has been designed to investigate the possible relationship between the vitamin D level in individual follicles and the oocyte developmental competence. Therefore, the first follicle from each ovary was aspirated separately, and follicular fluid (FF) from that follicle was matched to its oocyte. Subsequently, we have followed the outcome of each oocyte, evaluating its ability to undergo fertilization, subsequent preimplantation embryonic development, implantation, and ultimately pregnancy.

## Materials and methods

### Patient population and study design

The study protocol was approved by the Ethics Committee of Pomeranian Medical University (PMU), Szczecin, Poland (ethical authorization number: KB-0012/90/10). All patients signed an informed consent approved by the PMU Ethics Committee. All methods were performed in accordance with the relevant guidelines and regulations of European Society of Human Reproduction and Embryology (ESHRE), The American Society for Reproductive Medicine (ASRM) and The Polish Society of Reproductive Medicine and Embryology (PSRME).

### Subjects

All infertile women without hydrosalpinx between the ages of 18 and 38 years were considered eligible. They were scheduled for controlled ovarian hyperstimulation (COH) in either agonist or antagonist protocol, intracytoplasmic sperm injection (ICSI) and a single embryo transfer (SET) within a period of 21 months (February 2014–October 2015). The primary aim of the study was to evaluate a possible relationship between the vitamin D level in FF and the matched oocyte’s ability to generate an embryo resulting in pregnancy and ultimately a delivery after ICSI. None of the patients in the study received vitamin D supplementation before COH. To avoid the potential negative impact of sperm quality on the ability to fertilize an egg or embryo development, couples with moderate or severe male factor were excluded. The exclusion criteria were as follows: (1) moderate (concentrations 5 million–10 million sperms/ml) and severe (concentrations less than 5 million sperms/ml) male factor, (2) poor ovarian response to gonadotropin stimulation defined as ≤ 3 retrieved oocytes, and (3) inability to retrieve an oocyte from the first aspirated follicle of both ovaries.

### 25(OH)D measurement

25-Hydroxyvitamin D is the primary circulating form of vitamin D and remains stable throughout the menstrual cycle [25]. Due to its stability, serum 25(OH)D concentration is viewed as the best indicator of vitamin D status [26, 27]. Li-heparin serum samples and follicular fluid samples from all patients were obtained on the day of oocyte retrieval and kept frozen at − 80 °C until the measurement was taken. 25(OH)D level was measured using the chemiluminescent immunoassay (CLIA) LIAISON® 25 OH Vitamin D TOTAL Assay (REF 310600), DiaSorin Inc., Stillwater, MN, USA. The limit of quantitation of the assay was ≤ 4.0 ng/ml. Precision, as reported in the assay instructions for use, was determined by testing six serum samples, and two levels of LIAISON 25 OH Vitamin D TOTAL controls over 20 days according to the CLSI protocol EP05-A2 [26]; the total precision coefficients of variation were in the range of 12.6–10.8% (7.9–112.1 ng/ml) for serum and 9.7–9.5% (18.0–61.8 ng/ml) for kit controls. Vitamin D deficiency was defined by the Institute of Medicine (IOM) and the Endocrine Society Clinical Practice Guidelines [28, 29] as serum 25(OH)D level < 20 ng/ml. Follicular fluid vitamin D norm remains unknown.

### Follicular fluid collection

FF samples were collected according to the strict procedure described in detail elsewhere [30]. To collect clear follicular fluid and to avoid multiple vaginal punctures and the numerous flushing of the needle with culture medium after every follicular puncture, as well as to minimize the risk of vaginal bleeding, we decided to include in the study FF only from the first nearest (available) aspirated (lead) follicle of each ovary. Consequently, vitamin D levels were representative for the lead follicle only, and not for the entire cohort of follicles from any given patient. Each ovarian follicle was aspirated independently and collected in a separate test tube to identify the matched single cumulus–oocyte–complex (COC). This approach was chosen to avoid cross-contamination from the flush medium or the FF of other follicles. Test tubes with more than one COC were excluded from the study. In each case collected, FF was checked afterward for red blood cells. Fluids with the red blood cells were disqualified. FF samples with matched mature metaphase II (MII) oocytes were centrifuged at 10,000×*g* for 10 min, and the supernatants were aliquoted and stored at − 80 °C.

### Intracytoplasmic sperm injection

This study included only women undergoing ICSI to allow a precise cumulus–oocyte–complex evaluation, especially with regard to its maturity/degradation prior to fertilization. Furthermore, during conventional IVF insemination, there are significant events that take place, such as sperm penetration through layers of supporting granulosa cells, sperm membrane breaching, and fusion; thus, IVF requires competent oocyte and capable spermatozoa. All these events are circumvented by the ICSI procedure. While ICSI may affect the integrity of an oocyte, the literature supports the concept that oocyte ICSI degeneration is operator-independent [31].

During ICSI micromanipulation, holding pipettes (K-HPIP-3335, Cook, USA) and injection pipettes (K-MPIP-1035, Cook, USA) were used. Injected oocytes were placed in Sydney IVF Cleavage Medium G20720, K-SICM-20 (Cook Medical Inc., Bloomington, IN, USA) immediately after the procedure. This medium was used during the first 72 h of the culture, and for the following 48 h, Sydney IVF Blastocyst Medium G20722, K-SIBM-20 (Cook Medical Inc., Bloomington, IN, USA) was used. Post-fertilization embryo culture was carried out in a single microdroplet (0.03 ml) of the medium on a Petri dish under a layer of mineral oil. Each fertilized oocyte was cultured in an individual drop.

### Embryo assessment

Embryos underwent regular embryo assessment as described by the 2011 Istanbul consensus [32]. A top quality embryo (TQE) on the second day of the culture was defined as an embryo with four symmetrical, non-fragmented blastomeres, whereas a TQE on the third day of the culture was defined as an embryo with eight symmetrical, non-fragmented blastomeres. On the fifth day of the culture, at the blastocyst stage, three criteria were taken into consideration: the development of inner cell mass (ICM), the appearance of trophectoderm (TE), and expansion of the blastocyst cavity. The TQE on day 5 was considered to have fully expanded (grade 4) through to a hatched (grade 5) blastocyst with a high-quality ICM (grade 1) and TE (grade 1) [32].

### Embryo transfer policy

In each case, a single embryo transfer was performed. Embryos were selected for fresh transfer on day 3 if there were one or two TQE in the whole group of embryos. In the presence of more than two TQE available in a cohort of developing embryos, day 5 ET was performed.

### Outcome measures

The primary aim of this study was to determine whether local-intrafollicular vitamin D correlates with ICSI/SET results. In particular, in order to examine development competence of oocyte, the following parameters were taken into consideration:

- Ability of the oocyte to form two pronuclei (2PN) zygote after ICSI
- Stages of embryogenesis based on accurate cell division and cellular differentiation of the embryo within fixed times
- Positive hCG rate after SET, measured 3 weeks after oocyte retrieval
- Clinical pregnancy rate after SET defined as the presence of an intrauterine sac with an embryonic pole demonstrating cardiac activity at 7 weeks of gestation
- Live birth rate after SET

We also investigated the association between vitamin D serum level and IVF clinical outcomes, such as chemical pregnancy rate, clinical pregnancy rate, and live birth rate.

### Statistical methods

To facilitate sample size, we decided to apply a study from Ozkan et al. [15] in which the difference in FF 25(OH)D between the women who achieved pregnancy and those of women that did not become pregnant were significantly different: 34.42 ± 15.58 vs. 25.62 ± 10.53, *P* = 0.013, respectively. Therefore, for two independent study groups using clinical pregnancy as a primary outcome using a significance level of 0.05 and a 95% confidence interval (CI), total sample size of 106 has a 90% power to detect the wanted difference. In our study, we included a group of 106 patients undergoing single embryo transfer (SET). Continuous variables are presented as means ± standard deviations, and categorical variables are presented as percentages. The analysis was performed with the use of an independent *t* test for continuous variables and with the use of the *χ*^2^ test for categorical variables. Stepwise logistic regression was applied to identify independent variables associated with miscarriage and live birth rates. The significance level of each candidate predictive variable was set at *P* < 0.05 to enter the model. After selection of the candidate predictive factors, the final model included those prognostic factors with statistical significance according to the Wald statistic test at a threshold of *P* < 0.05. Receiver operating characteristics (ROC) curves were also fitted to obtain cutoff values for vitamin D levels using the area under the curve (AUC). Analyses were performed using Statistica 10 software (StatSoft Inc., USA).

## Results

### Study group characteristics

Out of 325 patients eligible for the study between February 2014 and October 2015, 198 patients were included in this study with a mean (± SD) age of 34.3 ± 3.9 and BMI of 23.15 ± 3.7 kg/m^2^ (Table 1). The indication for ICSI was primarily an idiopathic infertility factor. Two thirds of the specimens were obtained from patients undergoing their first intracytoplasmic sperm injection/single embryo transfer (ICSI/SET). Almost half of the women underwent COH in GnRH agonist protocol. In 124 patients, FF was obtained from both ovaries, whereas in 74 patients, FF was obtained from one ovary. In 106 patients, transferred embryos developed from oocytes with matched FF (all data are in Table 1).

**Table 1:** Characteristics of patients and ovarian stimulation

Patients characteristics	
Number of patients	198
Age [years], mean (SD)	34.3 (3.9)
BMI [kg/m^**2**^], mean (SD)	23.15 (3.7)
Caucasian ethnicity, *n* (%)	198 (100)
Duration of infertility [years], mean (SD)	3.4 (1.5)
Type of infertility, *n* (%)^a^	
Primary infertility	162 (81.8)
Mild male factor	20 (10.1)
Idiopathic	123 (62.1)
Ovulatory disorders	27 (13.6)
Tubal factor	18 (9)
Endometriosis	24 (12.1)
Previous IVF/ICSI attempts, *n* (%)	
0	148 (74.7)
≥ 1	50 (25.3)
Stimulation protocol characteristics	
Basal AMH [ng/ml], mean SD	2.74 (1.75)
Basal FSH [mIU/ml], mean SD	7.51 (4.12)
Type of GnRH analog	
Antagonist, *n* (%)	107 (54)
Agonist, *n* (%)	91 (46)
Gonadotrophins [IU], mean (SD)	2177.6 (893.8)
Estradiol on the day of hCG [pg/ml], mean (SD)	2392.2 (1357.8)
Progesterone on the day of hCG [ng/ml], mean (SD)	0.918 (0.4)
Follicular fluid samples characteristics	
TVS diameter of aspirated follicle [mm], mean (SD)	19.9 (1.8)
Volume of aspirated follicle fluid [ml], mean (SD)	3.7 (1.9)
Number of identified MII oocytes, *n*	322
Patients with FF samples from both ovaries, *n*	124
Patients with FF sample only from one ovary, *n*	74
Vitamin D3 level in follicular fluid [ng/ml], mean (SD)	29.6 (13.1)
Embryological outcome of the oocytes with matched FF	
Fertilization rate (%), mean (SD)	78.3 (41)
Percentage of TQE (%), mean (SD)	16.7 (37)
Number of SET with an embryo developed from FF matched oocyte^b^, *n*	106
SET of TQE^c^, *n*	54
Embryo cryopreservation^d^, *n*	60

*BMI* body mass index, *hCG* human chorionic gonadotropin, *TQE* top quality embryo, *TVS* transvaginal ultrasound*, SET* single embryo transfer

^a^The sum is greater than the total number of patients (198) since some couples met more than one indication for infertility treatment

^b^Among 198 patients 106 had SET of embryo developed from the oocyte with matched follicular fluid

^c^*n* = 54 cases transferred embryo was scored as TQE

^d^In 60 cases oocyte with matched follicular fluid produced an embryo suitable for cryopreservation

### Follicular fluid vitamin D level and embryological data

**Fig. 1:**
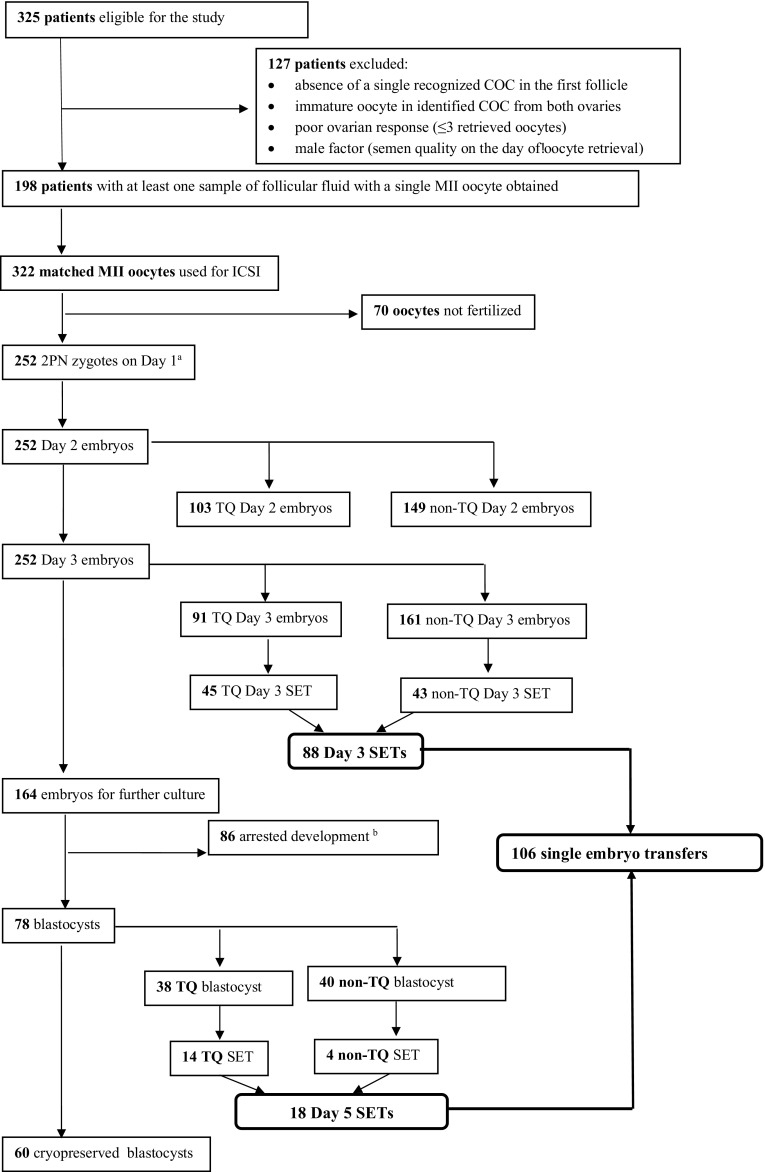
Patient selection process and flowchart of oocyte and subsequent embryo development. COC cumulus–oocyte–complex, MII oocyte metaphase II oocyte, ICSI intracytoplasmic sperm injection, TQ top quality, non-TQ non-top quality. ^a^Embryo grading details are presented in the “Materials and methods” section. ^b^Arrested development refers to embryos that failed to progress from day 3 to the blastocyst stage

**Fig. 2:**
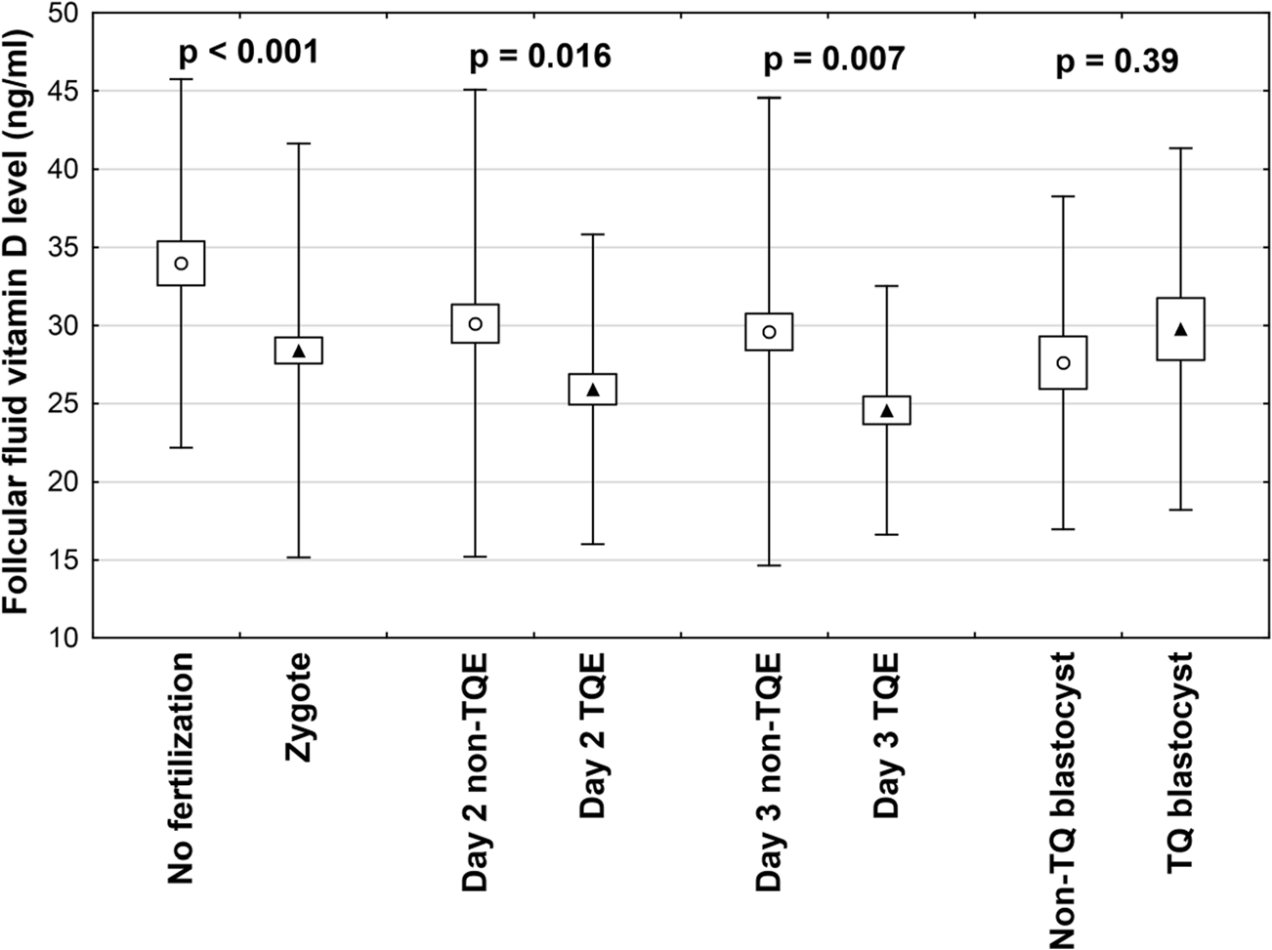
Vitamin D concentrations in FF in relation to the outcome of corresponding oocytes: fertilization and subsequent early embryo development. Each bar represents mean ± SD. FF follicular fluid, TQE top quality embryo, non-TQE non-top quality embryo. Embryo grading details are provided in the “Materials and methods” section

Out of total collected 322 FF samples, 74 were obtained from one ovary of a patient and 248 from both ovaries of 124 patients. We found a positive correlation between serum and follicular fluid 25(OH)D levels (*r* = 0.721, *P* < 0.001). The intrafollicular concentrations of 25(OH)D in the FF from the same patient varied and correlated with oocyte fate after ICSI. Mean diameter of aspirated follicle was 19.9 ± 1.8 mm and mean volume of aspirated follicle fluid 3.7 ± 1.9 ml (Table 1). Key stages of embryo development are presented in a flowchart in Fig. 1. Vitamin D levels were significantly lower in FF matched to oocytes that were successfully fertilized after ICSI compared to those that were not fertilized (28.4 ± 13.2 vs. 34.0 ± 11.8 ng/ml, *P* = 0.001) (Fig. 2). Furthermore, top quality (TQ) embryos on the second and third day after ICSI were produced from oocytes obtained from follicles with significantly lower vitamin D levels (25.9 ± 9.9 vs. 30.1 ± 14.7 ng/ml, *P* = 0.016 and 24.56 ± 7.9 vs. 29.59 ± 14.8 ng/ml, *P* = 0.007, respectively). There was no difference in FF vitamin D levels between the TQE and non-TQE blastocyst (25.8 ± 9.0 vs. 26.3 ± 8.6, ng/ml *P* = 0.39).

### Follicular fluid vitamin D level and clinical outcomes

**Fig. 3:**
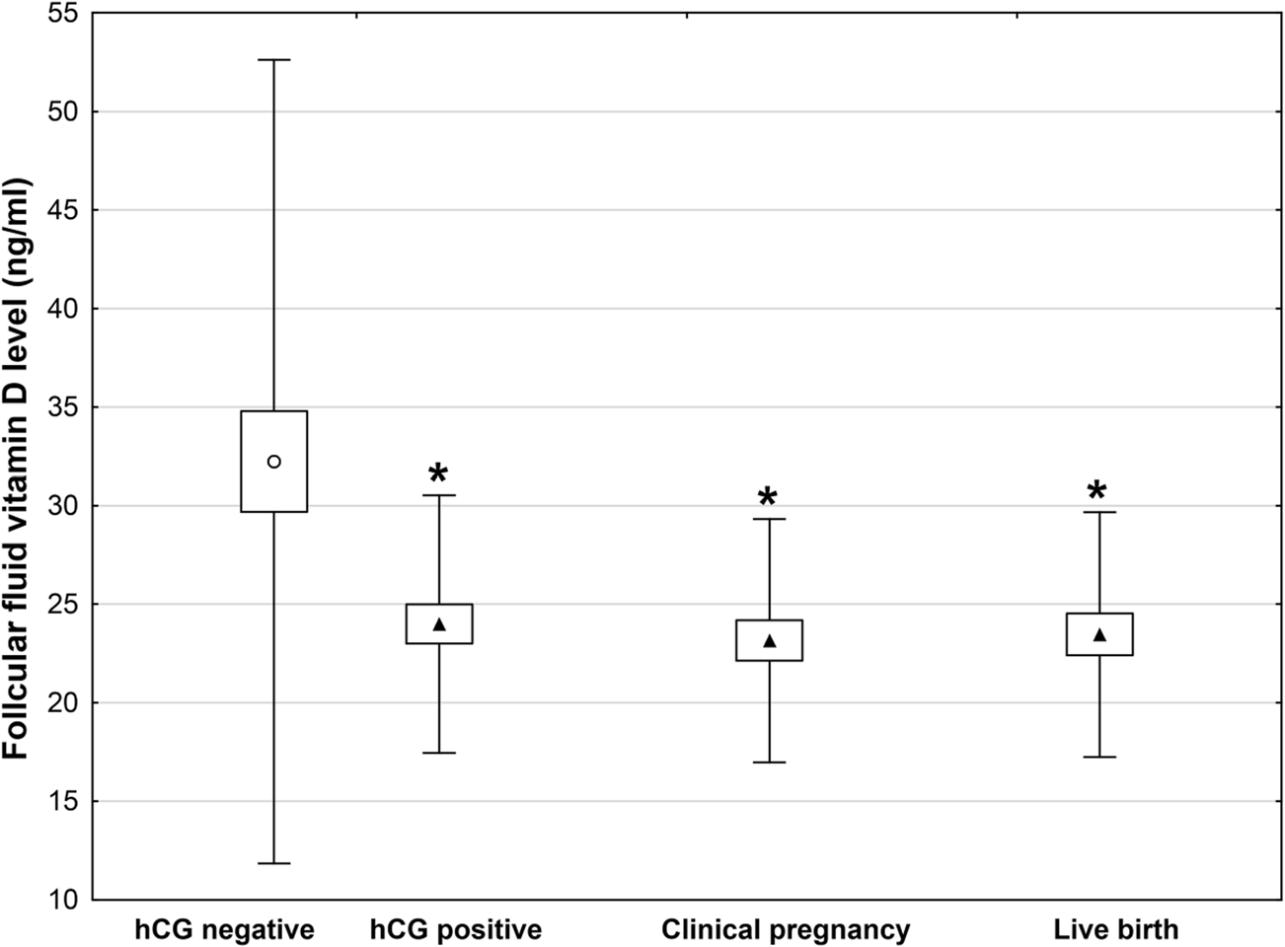
Vitamin D levels in FF corresponding to oocytes that led to development of transferred embryos and subsequent clinical outcomes. Each bar represents mean ± SD; *P < 0.001 vs. negative hCG following SET. FF follicular fluid, SET single embryo transfer

In 106 SETs, 88 embryos were transferred on the third day, and the remaining 18 were transferred on the fifth day of the culture (Fig. 1). A positive pregnancy test was detected in 42/106 (39.6%) of the patients, while clinical pregnancy and live birth rates in 36/106 (34%) and 34/106 (32.1%), respectively. As shown in Fig. 3, positive hCG, clinical pregnancy, and live birth rates were achieved from embryos derived from oocytes that grew in FF with significantly lower 25(OH)D levels than in follicles not associated with subsequent pregnancy (*P* < 0.001). Specifically, the concentration of 25(OH)D in FF in women with negative hCG was 32.23 ± 20.21 ng/ml, positive hCG 23.62 ± 6.09 ng/ml, clinical pregnancy 23.13 ± 6.09 ng/ml, and live birth 23.45 ± 6.11 ng/ml (*P* < 0.001).

### Serum vitamin D level and ICSI outcomes

Table 2 compares outcomes from patients with and without vitamin D deficiency according to the Institute of Medicine (IOM) and the Endocrine Society clinical practice guidelines [28, 29]. No significant differences were observed between these two groups concerning age, BMI, duration and type of infertility, the number of retrieved oocytes, the number of mature MII oocytes, the number of cycles with no available embryo for ET, the percentage of top quality embryos, or the quality of transferred embryo and cycles with elective SET (eSET). However, women with a vitamin D deficiency had a significantly higher fertilization rate compared to women with normal vitamin D levels (71 vs. 61.6%, *P* = 0.026) (Table 2). The incidence of vitamin D deficiency did not vary significantly across the seasons.

**Table 2:** Embryo development and clinical outcomes according to serum vitamin D serum status

	Vitamin D < 20 ng/ml	Vitamin D ≥ 20 ng/ml	*P* value
Patients’ characteristics			
Number of patients	114	84	
Age [years], mean (SD)	34.3 (4.1)	34.25 (3.7)	0.810^e^
Vitamin D status [ng/ml], mean (SD)	14.53 (3.7)	25.08 (4.0)	0.001^e^
Number of PCOS patients, *n* (%)	18 (15.8)	9 (10.7)	0.186^f^
PCOS vitamin D status [ng/ml], mean (SD)	12.32 (3.2)	24.03 (3.3)	0.001^e^
BMI [kg/m^2^], mean (SD)	22.89 (3.6)	23.51 (3.9)	0.734^e^
Number of obese patients, *n* (%)	26 (22.8)	20 (25)	0.826^f^
Obese vitamin D status [ng/ml], mean (SD)	14.1 (3.5)	25.6 (5.5)	0.001^e^
Duration of infertility [years], mean (SD)	3.3 (1.6)	3.5 (1.3)	0.792^e^
Type of infertility, *n* (%)^a^			
Primary infertility	93 (81.5)	69 (82.1)	0.444^f^
Mild male factor	11 (9.6)	9 (10.7)	0.473^f^
Idiopathic	71 (62.3)	52 (61.9)	0.605^f^
Ovulatory disorders	15 (13.1)	12 (14.2)	0.251^f^
Tubal factor	10 (8.7)	8 (9.5)	0.639^f^
Endometriosis	14 (12.3)	10 (11.9)	0.488^f^
Previous IVF/ICSI attempts, *n* (%)			
0	86 (75.4)	63 (75)	0.428^f^
≥ 1	28 (24.6)	21 (25)	
Stimulation protocol characteristics			
Basal AMH [ng/ml], mean SD	2.84 (1.56)	2.60 (1.98)	0.271^e^
Basal FSH [mIU/ml], mean SD	6.97 (4.27)	7.83 (4.09)	0.103^e^
Type of GnRH analog			
Antagonist, *n* (%)	59 (51.7)	48 (57.1)	0.175^f^
Agonist, *n* (%)	55 (48.2)	36 (42.9)	
Gonadotrophins [IU], mean (SD)	2125 (657)	2242 (1121)	0.694^e^
Estradiol on the day of hCG [pg/ml], mean (SD)	2493 (1326)	2263 (1394)	0.259^e^
Progesterone on the day of hCG [ng/ml], mean (SD)	0.93 (0.43)	0.89 (0.36)	0.373^e^
Embryological outcome			
Number of oocytes retrieved, mean (SD)	10.01 (4.9)	8.86 (3.9)	0.075^e^
Number of MII oocytes, mean (SD)	6.28 (2.7)	6.16 (2.6)	0.752^e^
Number of MI oocytes, mean (SD)	2.08 (1.9)	1.71 (1.6)	0.152^e^
Number of GV oocytes, mean (SD)	1.52 (1.9)	1.11 (2.1)	0.163^e^
Fertilization rate (%), mean (SD)	71 (29)	61.6 (29)	0.026^e^
Cycles with no available embryo for ET, *n* (%)	4 (3.5)	4 (4.8)	0.489^e^
Percentage of top quality embryos (%), mean (SD)	23.8 (22.4)	19.8 (21.2)	0.209^e^
Single embryo transfer^a^, *n* (%)	110 (96.5)	80 (95.2)	0.423^f^
Elective SET (eSET)^b^, *n* (%)	57 (51.8)	41 (51.2)	0.683^f^
Top quality transferred embryo, *n* (%)	67 (60.9)	43 (53.7)	0.235^f^
Season of embryo transfer, number of patients (%)	110	80	
Winter	5 (4.5)	3 (3.7)	0.073^g^
Spring	39 (35.5)	20 (25)	
Summer	55 (50)	47 (58.8)	
Autumn	11 (10)	10 (12.5)	
Clinical outcomes			
Positive hCG, *n* (%)^c^	57 (51.8)	23 (28.7)	0.001^f^
Clinical pregnancy, *n* (%)	53 (48.2)	20 (25)	0.001^f^
Miscarriage, *n* (%)^d^	16 (14.5)	3 (3.8)	0.013^f^
Live birth, *n* (%)^b^	41 (37.3)	20 (25)	0.067^f^

^a^In eight cases, there was no ET and these patients were not included in the live birth analysis

^b^Elective single transfer (eSET) represents a transfer of one embryo from a larger cohort of available day 3 or 5 embryos suitable for embryo transfer

^c^Positive hCG rate after SET, measured 3 weeks after oocyte retrieval

^d^Miscarriage was defined as any pregnancy loss after the pregnancy was confirmed 3 weeks after retrieval by a positive beta-hCG. This corresponds to 5 weeks of gestation when the gestational sacs on the US can be visualized.

^e^Student’s *t* test

^f^Chi-square test

^g^Fisher’s exact test

Overall, 42.1% of patients had a positive pregnancy test, 38.4% a clinical pregnancy, and 32.1% had a live birth. When analyzing the results according to 25(OH)D serum levels, we found that patients with a vitamin D deficiency had significantly higher clinical pregnancy rates compared to those with 25(OH)D values exceeding 20 ng/ml (48.2 vs. 25%, *P* = 0.001). However, serum vitamin D-deficient patients had also significantly higher miscarriage rates (14.5 vs. 3.8%, *P* = 0.013). The difference in the live birth rates between these groups did not reach statistical significance (37.3 vs. 25%, *P* = 0.067). According to ROC, serum vitamin D had high sensitivity and specificity for miscarriage with a cutoff value of 16.9 ng/ml and AUC = 0.78, *P* = 0.001 (Table 3).

**Table 3:** Receiver operating characteristic curve for vitamin D serum status

	Cutoff value (ng/ml)	AUC	SE	95% CI		*z*	*P*
Availability of top quality embryo	12.7	0.56	0.03	0.50	0.62	1.87	0.06
Miscarriage	16.9	0.78	0.03	0.71	0.84	8.53	< 0.01
Live birth	20.3	0.57	0.03	0.50	0.63	2.03	0.04

*AUC* the area under the ROC curve, *SE* standard error, *95% CI* 95% confidence intervals, *z z* score

Logistic regression analysis identified the following variables as associated with miscarriage: vitamin D deficiency, number of retrieved oocytes, and availability of TQEs. Normal vitamin D serum levels (≥ 20 ng/ml) were associated with lower miscarriage rates, with an odds ratio (OR, 95% CI) of 0.88 (0.80–0.97) (*P* = 0.01). The predictors of live birth were as follows: age, vitamin D deficiency, progesterone elevation on the day of hCG, number of retrieved oocytes, and availability of TQEs. Notably, women with a vitamin D deficiency had slightly higher live birth rates with an OR (95% CI) of 1.09 (1.04–1.15), (*P* = 0.01) than women with normal vitamin D levels (Table 4).

**Table 4:** Logistic regression models

	*B* ^a^	SE	Wald *χ*^2^	*P* value	OR^a^	95% CI
Miscarriage						
Vitamin D serum level ≥ 20 ng/ml	− 0.12	0.05	6.43	0.01	0.88	0.80–0.97
Number of oocytes retrieved	− 0.25	0.09	7.80	0.01	0.77	0.64–0.93
TQEs not available	1.33	0.37	12.67	0.01	3.80	1.81–7.96
Live birth						
Age	0.22	0.05	21.93	0.01	1.25	1.14–1.38
Vitamin D serum level < 20 ng/ml	0.09	0.03	10.76	0.01	1.09	1.04–1.15
Number of oocytes retrieved	0.45	0.09	23.89	0.01	1.58	1.31–1.90
TQEs available	1.09	0.20	29.49	0.01	8.89	4.04–19.56
Progesterone elevation on the day of hCG	− 0.07	0.03	4.19	0.04	0.93	0.86–0.99

*B* regression coefficient, *SE* standard errors of regression coefficient, *OR* odds ratios, *95% CI* 95% confidence intervals

^a^*B* < 0 and OR < 1 mean miscarriage or live birth is inclined to be negative

## Discussion

IVF provides a unique opportunity to study the relationship of both systemic and intrafollicular levels of vitamin D with the fate of individual oocytes since it is possible to track follicular development, oocyte fertilization, embryo development, implantation, and ultimately, clinical outcome. To date, the relationship between the level of vitamin D and IVF outcome(s) remains controversial [27, 33, 34]. Available systematic reviews and meta-analyses of serum vitamin D and IVF outcomes do not support the idea of routinely screening 25(OH)D serum status to predict the clinical pregnancy rate, nor supplementing vitamin D in couples undergoing ART [27, 33, 34]. In this study, 25(OH)D serum deficiency was associated with a slightly higher odds of life birth despite a higher miscarriage rate. Hence, it appears that the serum level of vitamin D has a complex relationship with clinical outcomes. Furthermore, present findings differ from the conclusions of several reviews on this subject, whereby preponderance of evidence suggested that a lower serum vitamin D was associated with lower live birth rates [27, 33, 34]. However, it is important to note that in the current study, the level of vitamin D was determined only on the day of oocyte pick-up. Vitamin D supplementation during pregnancy is associated with increased circulating 25(OH)D levels, birth weight, and birth length [35].

Consistent with our observations, previous studies (using pooled FFs) have demonstrated a significant correlation of serum and FF levels of 25(OH)D noting also that the mean vitamin D concentration was higher in FF than serum [13, 15, 18, 23]. These observations indicate that peripheral vitamin D status is a reliable indicator of 25(OH)D availability within the ovary. The mechanisms of action by which vitamin D may affect oocyte competence in IVF remain unclear. By evaluating vitamin D levels in a large number of individual follicles and monitoring the outcome of their corresponding oocytes, the present study demonstrates for the first time that local-intrafollicular vitamin D levels strongly and negatively correlate with the quality of the oocyte. Based on these observations, we propose that intrafollicular concentration of vitamin D may be viewed as a marker of oocyte quality. Furthermore, one can speculate that the local and yet not characterized actions of vitamin D may contribute to oocytes’ ability to be successfully fertilized, develop into an embryo, and ultimately affect the likelihood of pregnancy in an infertile population undergoing ICSI/SET. Potential explanations for these findings may invoke either direct or indirect action of vitamin D on pathways involved in the regulation of follicle/oocyte maturation [9, 36, 37].

Previous studies examining 25(OH)D FF levels and IVF outcomes yielded conflicting results. Ozkan et al. [15] reported a positive association between FF 25(OH)D levels and IVF outcomes, with patients in the highest tertile of FF 25(OH)D distribution being almost fourfold more likely to achieve a clinical pregnancy rate (CPR) compared to patients in the lowest tertile. In contrast, Anifandis et al. observed a negative relationship between increasing FF vitamin D levels and IVF outcomes [23]. When comparing the FF pooled from all retrieved follicles between patients with a serum vitamin D level less than 20 ng/ml and to those with 21–30 and > 30 ng/ml, a significantly higher mean score of embryo quality, higher CPR per women and higher CPR per ET was found in patients with a vitamin D deficiency [23]. Subsequently, Aleyasin et al. demonstrated decreased fertilization rates with increasing tertiles of 25(OH)D level in follicular fluid, showing a potentially negative association between 25(OH)D and oocyte competence [18]. However, these data are difficult to interpret because all of the women in this population, except for one, were vitamin D deficient [18]. Recently, Firouzabadi et al. divided patients according to vitamin D serum levels into three groups: deficient, insufficient, and sufficient defined as < 10, 10–29, and 30–100 ng/ml, respectively [13]. In contrast to the reports by Anifandis et al. and Aleyasin et al., the fertilization rates and implantation rates were related to vitamin D levels. Furthermore, good-quality embryos in the serum deficient, insufficient, and sufficient groups were also found at comparable rates of 82.6, 90.2, and 86.7%, respectively. However, the level of vitamin D in the FF of the pregnant group was 22.17 ± 17.21 ng/ml compared with 26.14 ± 19.33 ng/ml in the non-pregnant group. The lack of statistical significance (*P* = 0.17) in that study could be related to the sample size, as only 7% of women were vitamin D sufficient in that studied population.

Our study has several strengths. Its primary strength pertains to the study design, whereby we were able to follow the fate of individual oocytes from fertilization through the assessment of embryo development to implantation and ultimately to live birth by evaluating patients who underwent a single embryo transfer. In contrast, previous reports included patients with an average of two to four transferred embryos [13, 15, 18, 23, 24]. Consequently, in this study, we were able to assess the direct relationship of FF vitamin D level with the development of a particular embryo and subsequent pregnancy. Another strength of the current study is the relatively high number of participants. Previous reports evaluated 80–101 patients [15, 18, 23, 24], while we prospectively assessed 198 participants. Furthermore, our population was homogeneous whereby all subjects were Caucasian. Previously published reports evaluated various populations such as Iranian [13, 18, 24], Greek [23], and highly diverse American patients including 53% Caucasian, 20% Asian, 16% Hispanic, and 7% African-American subjects [15]. The issue of race is relevant since Rudick et al. demonstrated that race affects the relationship between vitamin D status and pregnancy rates after IVF [16]. For example among non-Hispanic whites, pregnancy rates declined with progressively lower levels of vitamin D, while among Asians, the reverse was observed.

To focus on the evaluation of oocyte competence, we excluded couples with moderate and severe male factor and therefore minimized the role of spermatozoa as a factor affecting embryo development. In contrast, in some previous studies, male factor was present in 58–65% of couples [13, 24] while in other studies [15, 18, 23], the indications for IVF/ICSI were not mentioned. Additionally, to test only mature oocytes, we evaluated only patients undergoing ICSI.

This study has several limitations. First, a further generalization of our findings will require testing of women from populations excluded from the present study. Second, this study evaluated vitamin D levels from the lead follicle only. Third limitation pertains to the nature of the observational study and consequently demonstration of an association but not causation with regard to the relationship between vitamin D level and biological outcomes. Demonstration of causation will require new interventional studies. Lastly, we did not measure vitamin D binding globulin level (VDBP). Therefore, in our study, we consider a total 25(OH)D level, as bioavailable 25(OH) D is defined by circulating 25(OH)D not bound to VDBP, which is analogous to the definition of bioavailable testosterone [38]. In humans, common genetic polymorphisms produce three major circulating variants of the VDBP (Gc1F, Gc2, and Gc1S) [39]. The phenotypic variations in the VDBP amino acid sequence are distinguished by single nucleotide polymorphisms (SNPs) rs7041 and rs4588. In 1993, Constans and Arnaud showed differences between VDBP polymorphisms, where the affinity of Gc1F was four times higher than that of Gc2 and double that of Gc1S [40]. Blacks and Asians are more likely to carry Gc1F, which has the highest affinity for 25(OH)D and is associated with low VDBP levels. Whites are more apt to carry Gc1S while Gc2, which has a lower affinity for 25(OH)D and is associated with higher VDBP levels, is frequently found in whites and rarely found in blacks. The high prevalence of Gc1F in blacks results in concentrations of bioavailable 25(OH)D similar to those in whites [41]. Therefore, bioavailable 25(OH)D may be a more appropriate cross-racial marker of vitamin D sufficiency [42]. It is worth stressing the fact that since levels of total 25(OH)D are, in part, genetically determined, our study is based on Caucasian population only.

Additionally, the issue of VDBP is particularly complicated because it is a highly polymorphic serum protein with three common alleles and more than 120 rare variants with different binding affinities [43]. Thus, standardization of such assays is currently problematic because the available calibration materials tend to contain either a single VDBP variant or a mix of variants of unknown composition [39]. Furthermore, great strides have been made in recent years to improve the accuracy of 25(OH)D assays, since a study done in 2012 used the same VDBP method adopted in the evaluation [43]. In our study, we have used up-to-date LIAISON Kit. In automated assays, 25(OH)D must be liberated from VDBP, and its rebinding prevented [39]. The assumption is that whatever approach is used, either 100% of the 25(OH)D is removed from the VDBP or, if not, the percentage removed is the same in all samples. In the Caucasian population, the most common VDBP phenotypes are Gc1S and Gc2 which have the lowest affinity for 25(OH)D [44].

Finally, recent study from Fabris et al. on oocyte donor population did not show any dependence or statistical association between the values of total 25(OH)D and bioavailable 25(OH)D with regard to the ovarian reserve, response to ovarian stimulation, oocyte quality in egg donors, or ongoing pregnancy rates after fresh embryo transfer in oocyte recipients [19].

Given the prevalence of vitamin D deficiency, which is estimated to affect approximately 1 billion people worldwide [1], the issue of the clinical impact of 25(OH)D on oocyte development is of great importance. The relationship between vitamin D and oocyte quality may be influenced by the bioavailability of vitamin D within an individual follicle. Recent data from rhesus monkey follicular cultures by Xu et al. [45] show that 1,25(OH)_2_D impacts follicular survival and growth, as well as oocyte growth in a dose- and stage-dependent manner. The low dose of 1,25(OH)_2_D promoted preantral follicular survival while a high dose led to greater follicular diameter. However, larger follicular size may not necessarily correspond to a higher quality of the oocyte. In our study, local-follicular fluid 25(OH)D concentration was strongly and negatively correlated with the oocyte quality. To reconcile these discordant observations, further studies should evaluate how follicular vitamin D may affect oocyte competence. Such studies may extend present findings and may provide a mechanistic explanation for the relationship of vitamin D and clinical outcomes. In the long term, local concentrations of vitamin D may become relevant to clinical decisions such as the selection of the best oocytes and therefore help to improve the results of IVF/ICSI.
